# RNA-Seq of Dermal Fibroblasts from Patients with Hypermobile Ehlers–Danlos Syndrome and Hypermobility Spectrum Disorders Supports Their Categorization as a Single Entity with Involvement of Extracellular Matrix Degrading and Proinflammatory Pathomechanisms

**DOI:** 10.3390/cells11244040

**Published:** 2022-12-14

**Authors:** Marco Ritelli, Nicola Chiarelli, Valeria Cinquina, Nicoletta Zoppi, Valeria Bertini, Marina Venturini, Marina Colombi

**Affiliations:** 1 Division of Biology and Genetics, Department of Molecular and Translational Medicine, University of Brescia, 25121 Brescia, Italy; 2 Division of Dermatology, Department of Clinical and Experimental Sciences, Spedali Civili University Hospital Brescia, 25121 Brescia, Italy

**Keywords:** cytokines, extracellular matrix, hypermobile Ehlers–Danlos syndrome, hypermobility spectrum disorders, inflammation, matrix metalloproteinases, myofibroblasts, RNA-seq

## Abstract

Hypermobile Ehlers–Danlos syndrome (hEDS) and hypermobility spectrum disorders (HSD) are clinically overlapping connective tissue disorders of unknown etiology and without any validated diagnostic biomarker and specific therapies. Herein, we in-depth characterized the cellular phenotype and gene expression profile of hEDS and HSD dermal fibroblasts by immunofluorescence, amplicon-based RNA-seq, and qPCR. We demonstrated that both cell types show a common cellular trait, i.e., generalized extracellular matrix (ECM) disarray, myofibroblast differentiation, and dysregulated gene expression. Functional enrichment and pathway analyses clustered gene expression changes in different biological networks that are likely relevant for the disease pathophysiology. Specifically, the complex gene expression dysregulation (mainly involving growth factors, structural ECM components, ECM-modifying enzymes, cytoskeletal proteins, and different signal transducers), is expected to perturb many ECM-related processes including cell adhesion, migration, proliferation, and differentiation. Based on these findings, we propose a disease model in which an unbalanced ECM remodeling triggers a vicious cycle with a synergistic contribution of ECM degradation products and proinflammatory mediators leading to a functional impairment of different connective tissues reflecting the multisystemic presentation of hEDS/HSD patients. Our results offer many promising clues for translational research aimed to define molecular bases, diagnostic biomarkers, and specific therapies for these challenging connective tissue disorders.

## 1. Introduction

The Ehlers–Danlos syndromes (EDS) represent a group of 14 heritable connective tissue disorders (HCTDs), the majority of which result from alterations in genes encoding collagens and enzymes involved in their biogenesis or in the extracellular matrix (ECM) homeostasis [1]. The most common form of these disorders is hypermobile EDS (hEDS) that is characterized by a marked variable phenotype and high rate of chronic disability and remains the unique variant without an identified molecular cause. Hence, hEDS is diagnosed only clinically based on a set of more stringent criteria presented in 2017 by the International EDS Consortium [2]. hEDS is currently defined by the simultaneous presence of generalized joint hypermobility (gJHM) according to an age-specific Beighton score (BS) together with a combination among at least 5 out 12 signs of multisystemic involvement plus either a positive family history and/or at least one musculoskeletal manifestation, and exclusion of other conditions with JHM (Table 1). Patients with symptomatic JHM not fulfilling these new criteria are nowadays characterized as having hypermobility spectrum disorders (HSD) [3].

Besides obvious consequences of JHM complications, both hEDS and HSD patients show numerous extra-articular manifestations not comprised in the current nosology. These JHM-associated comorbidities include chronic fatigue, pelvic floor problems, bladder dysfunction, increased susceptibility to osteoarthritis, various dysautonomic features (e.g., postural orthostatic tachycardia syndrome, gastrointestinal dysfunction), alterations of the immune system, headache/migraine, sleep disorders, behavioral disturbances, and psychological distress [1,2,3,4,5,6]. After the publication of the updated diagnostic criteria, several authors raised serious doubts about their limits, and it is still debated if hEDS and HSD are distinct clinical entities rather than part of a phenotypic spectrum that require a similar pattern of multidisciplinary intervention [7,8,9,10,11,12,13]. Considering the overlapping clinical presentation of hEDS and HSD patients and the absence of any objective and validated diagnostic biomarker, many researchers currently use the terms hEDS and HSD interchangeably and these two diagnostic labels are often grouped together as hEDS/HSD [10,11,12,13,14,15,16,17,18].

Over the last years, our group contributed to this current view by demonstrating that dermal fibroblasts from hEDS and HSD patients share a common cellular phenotype [19,20], which is not present in other EDS types and HCTDs [21]. Indeed, although in a small cohort of cell strains, we demonstrated that both patients’ cells show a combination of ECM disarray and myofibroblast differentiation sustained by an αvβ3 integrin-mediated signal transduction pathway involving the integrin linked kinase and the transdifferentiation factor Snail1. This peculiar pathologic phenotype can be induced in control fibroblasts by hEDS and HSD cell-derived conditioned media, in which distinct degradation fragments of collagen type I (COLLI-fs), fibronectin (FN-fs), and tenascins (TNs-fs) together with high amounts of ECM-degrading matrix metalloproteinases (MMPs) were identified [19,22].

This study was designed to confirm in a large cohort of hEDS and HSD dermal fibroblasts the peculiar pathologic cellular phenotype and to investigate whether these cells show a distinct or common gene expression profile. The comparison of patients’ transcriptome profiles to that of control fibroblasts by RNA-seq also aimed to provide significant insights in the pathobiology of these disorders.

## 2. Patients and Methods

### 2.1. Patients

The research cohort comprised 20 adult hEDS females, 20 adult HSD patients (16 females, 4 males), and 40 healthy donors (34 females, 6 males), who were clinically evaluated at the specialized outpatient clinic for Ehlers–Danlos syndromes and related connective tissue disorders of the University Hospital Spedali Civili of Brescia. Patients were diagnosed according to the 2017 EDS nosology [2] and healthy individuals were selected with an age range similar to that of patients and by excluding any clinical feature reported in the hEDS nosology, as well as several associated comorbidities.

A total of 6 hEDS (P1-P6), 3 HSD patients (H1-H3), and 12 healthy donors (C1-C12) were previously reported [19,22,23], whereas the other hEDS (P7-P20) and HSD (H4-H20) patients, and healthy donors (C13-C40) were novel. Among HSD, 8 females and 2 males were generalized HSD, since they fulfilled criterion 1 but were negative for criterion 2, even if they all showed musculoskeletal manifestations (feature C of criterion 2), 4 females and 1 male fulfilled criterion 2 for the combination feature A plus C but were negative for criterion 1, and 4 females and 1 male were negative for both gJHM and criterion 2 even with a positive feature C. A comprehensive description of patients’ nosologic criteria and additional mucocutaneous, osteoarticular, musculoskeletal, cardiovascular, gastrointestinal, uro-gynecological, neuropsychiatric, atopic, and ocular features is available in Supplementary Table S1.

Prior to RNA-Seq, in all patients, we performed an NGS panel covering almost all EDS-associated genes and those of some overlapping conditions [24] as well as *TNXB* and *AEBP1* Sanger sequencing [25,26], which did not identify any (likely) pathogenetic variant.

### 2.2. Skin Biopsies, Cell Cultures, and Immunofluorescence Microscopy (IF)

Skin biopsies derived from hEDS, HSD, and healthy individuals were all processed by the same standard protocol previously described [19]. Fibroblasts were grown between the 1st and 4th in vitro passage at 37 °C in a 5% CO_2_ atmosphere in Earle’s Modified Eagle Medium supplemented with 2 mM L-glutamine, 10% FBS, and 100 µg/mL penicillin and streptomycin (Thermofisher Scientific, Rodano, Italy). IF of the control, hEDS, and HSD cells investigating the expression and organization of COLLI, COLLIII, and COLLV, FN, β-actin, cadherin-11, α2β1, α5β1, and αvβ3 integrins, all isoforms of TN, α-SMA, and Snail1 were performed with specific antibodies and protocols, as previously described [19,22,23].

### 2.3. RNA Extraction and Transcriptome Sequencing

Total RNA was purified from patient and control cells grown for 72 h at 70–80% of confluency using the miRNeasy kit (Qiagen, Milano, Italy). Sample quality and quantity were evaluated with the Agilent 2100 Bioanalyzer (Agilent Technology, Cernusco sul Naviglio, Italy) and the Qubit fluorometer (Thermofisher Scientific, Rodano, Italy), respectively.

To improve statistical power, RNA samples from all experimental groups were sequenced twice as biological replicates (at the 2nd and 4th in vitro passage, respectively) plus 20 technical replicates for control fibroblasts and 10 for both hEDS and HSD cells.

Reverse transcription and library preparation were achieved following the standard protocol of the Ion Ampliseq Transcriptome Human Gene Expression Kit (Thermofisher Scientific, Rodano, Italy), which targets by multiplexed PCR 18,574 protein-coding and 2228 non-coding RNAs (mRNAs/ncRNAs). Libraries were quantified with the Ion qPCR Library Quantification Kit, diluted to 100 pM and pooled equally, with 8 samples per pool, followed by emulsion PCR and enrichment with the Ion OneTouch2 with ES system. Libraries were then loaded onto an Ion 540 chip and sequenced on the Ion S5 instrument.

Differentially expressed genes (DEGs) were identified with the Transcriptome Analysis Console 4.0 with the ampliSeqRNA plugin (Thermofisher Scientific, Waltham, MA, USA) and applying an ANOVA test with a fold-change threshold of ±1.5 and an FDR-adjusted *p*-value ≤ 0.01.

### 2.4. Functional Gene Annotation and Enrichment Analyses

DEGs were annotated based on Gene Ontology (GO) terms by querying the DAVID database. We also used the ClueGO (v. 2.5.9) and CluePedia (v. 1.5.9) plugins of the Cytoscape software (v. 3.9.1) to create and visualize functionally grouped networks by integrating the GO categories “Biological Process”, “Molecular Function”, and pathways from KEGG, Reactome, and WikiPathways databases.

We applied a two-sided hypergeometric test and only functional annotations showing a Benjamini–Hochberg corrected *p*-value ≤ 0.01 were considered by applying default parameters, except for a minimum number of 3 genes and 7% of genes for GO terms and a kappa-score threshold = 0.7.

**Table 1:** hEDS diagnostic checklist listing the three mandatory criteria according to the 2017 nosology [2].

**CRITERION 1** Presence of generalized joint hypermobility (gJHM)
BEIGHTON SCORE
≥6 for prepubertal children and adolescents; ≥5 for pubertal men and women ≤ 50 years of age; ≥4 for men and women > 50 years of age. In individuals with acquired joint limitations (past surgery, wheelchair, amputations, etc.) affecting the Beighton score calculation, the assessment of GJH includes historical information using the following five-point questionnaire (5PQ) [27]. In these cases, if the Beighton score is 1 point below the age- and sex-specific cut-off and the 5PQ is ‘positive’ (at least two positive items), then a diagnosis of GJH can be made. Can you now (or could you ever) place your hands flat on the floor without bending your knees? Can you now (or could you ever) bend your thumb to touch your forearm? As a child, did you amuse your friends by contorting your body into strange shapes or could you do the splits? As a child or teenager, did your shoulder or kneecap dislocate on more than one occasion? Do you consider yourself ‘double-jointed’?
**CRITERION 2** Two or more among features (A–C) MUST be present (A and B; A and C; B and C; A and B and C)
➢ **Feature A (must have at least five):** Unusually soft or velvety skin; Mild skin hyperextensibility (>1.5 cm); Unexplained striae such as striae distensae or rubrae at the back, groins, thighs, breasts, and/or abdomen in adolescents, men or prepubertal women without a history of significant gain or loss of body fat or weight; Bilateral piezogenic papules of heels; Recurrent or multiple abdominal hernia(s) (e.g., umbilical, inguinal, crural); Atrophic scarring involving at least 2 sites and without the formation of truly papyraceous and/or hemosideric scars as seen in classical EDS; Pelvic floor, rectal, and/or uterine prolapse in children, men or nulliparous women without a history of morbid obesity or other known predisposing medical condition; Dental crowding and high or narrow palate; Arachnodactyly, as defined in one or more of the following: (i) positive wrist sign (Steinberg sign) on both sides; (ii) positive thumb sign (Walker sign) on both sides; Arm span-to-height ≥ 1.05; Mitral valve prolapse (MVP) mild or greater based on strict echocardiographic criteria; Aortic root dilatation with Z-score > +2.
➢ **Feature B:** Positive family history of hEDS with at least one first-degree relative independently meeting hEDS criteria.
➢ **Feature C (at least one of the following musculoskeletal manifestations):** Musculoskeletal pain in two or more limbs, recurring daily for at least 3 months; Chronic widespread pain for at least 3 months; Recurrent joint dislocations or frank joint instability in the absence of trauma.
**CRITERION 3** All the following prerequisites must be met:
Absence of unusual skin fragility, which should prompt consideration of other types of EDS. Exclusion of other heritable and acquired connective tissue disorders, including autoimmune rheumatologic conditions. In patients with an acquired connective tissue disorder (e.g., lupus, rheumatoid arthritis, etc.), additional diagnosis of hEDS requires meeting both Features A and B of Criterion 2. Feature C of Criterion 2 (chronic pain and/or instability) cannot be counted towards a diagnosis of hEDS in this situation. Exclusion of alternative diagnoses that may also include joint hypermobility by means of hypotonia and/or connective tissue laxity. Alternative diagnoses and diagnostic categories include, but are not limited to, neuromuscular disorders (e.g., myopathic EDS, Bethlem myopathy), other HCTD (e.g., other types of EDS, Loeys–Dietz syndrome, Marfan syndrome), and skeletal dysplasias (e.g., Osteogenesis imperfecta). Exclusion of these considerations may be based upon history, physical examination, and/or molecular genetic testing, as indicated.

### 2.5. qPCR

Relative expression levels of selected DEGs were confirmed by qPCR using different RNA extractions obtained from 10 randomly chosen hEDS, 10 HSD, and 20 control fibroblasts at the 3rd in vitro passage. 3 μg of pooled hEDS/HSD and control RNA samples were reverse transcribed with random primers and qPCR was performed by standard thermal cycling conditions using the QuantStudio 3 Real-Time PCR System (ThermoFisher Scientific, Rodano, Italy) as previously reported [20]. A subset of DEGs was also verified by qPCR pooling a total of 3 μg of RNA from all control, hEDS, and HSD cell strains also including their biological replicates and considering hEDS and HSD as separate groups.

## 3. Results

### 3.1. Comparison of the Cellular Phenotype of Dermal Fibroblasts Derived from hEDS and HSD Patients to That of Healthy Donors

**Figure 1:**
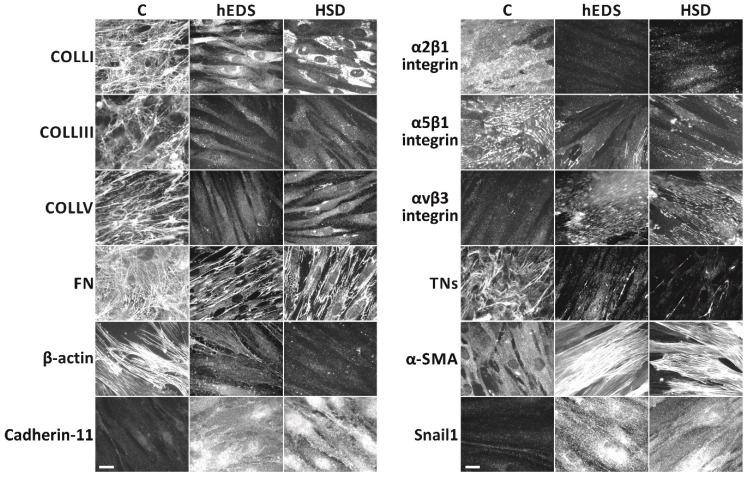
Widespread ECM disorganization and myofibroblast differentiation of hEDS and HSD cells. Control and patients’ cells grown on glass coverslips for 72 h were analyzed by IF with specific Abs directed against COLLI, COLLIII, COLLV, FN, TNs, α2β1, α5β1, and αvβ3 integrin receptors, β-actin, α-SMA, cadherin-11, and Snail1, as previously detailed [19,22,23]. The images are representative of 40 control and 20 hEDS and 20 HSD cell strains. Scale bar: 7 μm.

To confirm the peculiar cellular features of hEDS and HSD fibroblasts previously demonstrated on a small number of patients’ cells [19], all fibroblasts from the new engaged hEDS (P7-P20), HSD (H4-H20), and healthy individuals (C13-C40) were assessed by IF for ECM organization of COLLI, COLLIII, COLLV, FN, and TNs, distribution of the α2β1, α5β1, and αvβ3 integrins, organization of β-actin and α-SMA cytoskeleton, and expression of cadherin-11 and Snail1. As shown in Figure 1, compared to control fibroblasts and like the previously reported patients’ cells, all hEDS and HSD cells showed marked disorganization of the analyzed ECM structural components, abnormal expression of the α2β1, α5β1, and αvβ3 integrins along with the presence of a well-organized α-SMA cytoskeleton instead of that of β-actin, and expression of cadherin-11 and Snail1.

### 3.2. Global Analysis of mRNA/ncRNA Expression Profiles in hEDS and HSD Dermal Fibroblasts

Following the confirmation of this distinctive cellular trait in all hEDS and HSD fibroblasts as well as of the normal phenotype of the control cells, we performed whole-genome transcriptome profiling in these cell strains by sequencing a total of 200 libraries.

**Figure 2:**
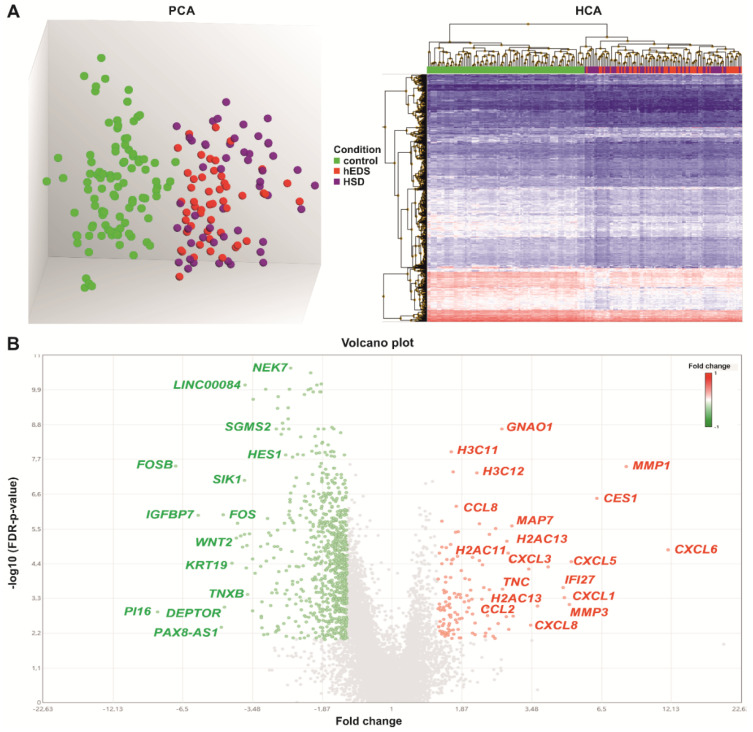
Differential gene expression and functional enrichment analyses. (**A**) On the left, PCA showing a clear distribution of control (green dots), hEDS (red dots), and HSD (violet dots) samples in two distinct clusters. On the right, HCA of the 952 DEGs identified in hEDS/HSD vs. control with downregulated genes in blue and upregulated ones in red. (**B**) Volcano plot displaying the distribution of the 826 downregulated (green) and the 126 upregulated (red) DEGs. The plots represent expression values as fold change (*x*-axis) plotted against the -log10 FDR-adjusted *p*-value (*y*-axis). Gene symbols for a selection of DEGs are indicated.

Differential gene expression analysis was initially performed comparing hEDS vs. control, HSD vs. control, and hEDS vs. HSD (Supplementary Table S2). In the former, a total of 722 DEGs were identified, whereas in HSD vs. control 1174 genes passed the filtering criteria. Remarkably, no DEGs were identified among hEDS and HSD, indicating a comparable gene expression profile that was confirmed also by principal component analysis (PCA), which showed two distinct clusters that clearly separate controls from patients but not hEDS from HSD which grouped together (Figure 2A).

Furthermore, by comparing the apparently different lists of DEGs identified in hEDS and HSD, 590 genes were not only common but also showed the same trend of differential expression (Supplementary Table S2). By analyzing the complete ANOVA tables of both groups, we recognized that the discrepancy in the number of DEGs (722 vs. 1174) was due to the stringent filtering criteria of differential gene expression analysis. Indeed, 132 DEGs present only in hEDS vs. control and 584 DEGs present only in HSD vs. control showed the same trend of differential expression in both groups, but they were lost either for an FDR > 1% and/or because they did not respect the fold change cut-off ± 1.5 (Supplementary Table S2).

Based on these findings, pointing out that hEDS and HSD cells share a common dysregulated gene expression pattern, we grouped hEDS and HSD within the same category and reanalyzed all data comparing hEDS/HSD vs. control, which revealed a total of 952 DEGs. As shown in the Volcano plot (Figure 2B), 826 DEGs were downregulated (750 mRNAs, 70 lncRNA, 6 snoRNA) and 126 showed an increased expression (122 mRNAs, 4 lncRNA). The full list of DEGs is available in Supplementary Table S2. In line with the PCA result, hierarchical clustering analysis (HCA) confirmed the presence of two distinct clusters of transcripts that plainly set apart hEDS/HSD from controls, as well as a significant overlap among the expression profiles of hEDS and HSD samples (Figure 2A, Supplementary Figure S1).

### 3.3. The DEGs in hEDS/HSD Are Involved in Biological Processes and Pathways That Are Potentially Relevant for the Disease Pathophysiology

**Figure 3:**
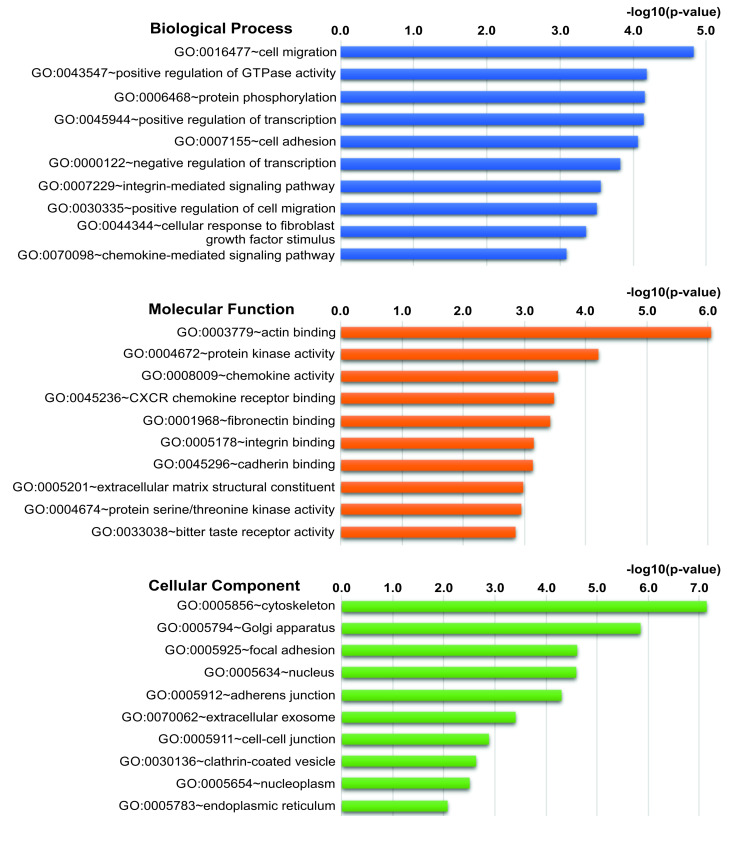
Gene Ontology functional enrichment analyses. Top ten enriched GO terms based on the categories “Biological Process”, “Molecular Function”, and “Cellular Component” by querying the DAVID database with the 952 DEGs identified in hEDS/HSD vs. control.

To obtain an overview about localization, function, and related biological processes of the protein products of the significantly enriched genes, we performed GO analysis on the full list of DEGs. Cell migration, positive regulation of GTPase activity, and protein phosphorylation (Biological Process); actin binding, protein kinase activity, and chemokine activity (Molecular Function); cytoskeleton, Golgi apparatus, and focal adhesion (Cellular Component) were the three most enriched GO terms for each category according to the DAVID database. The remaining top 10 GO terms and the complete GO results are available in Figure 3 and Supplementary Table S3.

Considering the disproportion between down- and upregulated genes (826 vs. 126), we performed GO analysis also separately. Positive regulation of transcription, protein phosphorylation, and cell migration were the most significant “Biological Process” terms for downregulated DEGs, while chemokine-mediated signaling pathway, neutrophil chemotaxis, and inflammatory response were the most enriched terms by the upregulated genes. 

The most significant “Molecular Function” categories for downregulated DEGs were actin binding, ATP binding, and protein serine/threonine kinase activity, whereas chemokine activity, structural constituent of chromatin, and CXCR chemokine receptor binding were that of the upregulated ones. Cytoplasm/cytoskeleton/cytosol, Golgi apparatus, and focal adhesion were the most enriched “Cellular Component” terms for downregulated genes, while nucleosome, extracellular region/space, and extracellular exosome were that of upregulated ones. The remaining top 10 GO terms for each category and the complete GO results are available in Supplementary Figures S2–S4 and Supplementary Table S4.

To uncover biological networks modulated by the 826 down- and 126 upregulated DEGs, we performed GO enrichment/depletion analysis combining both lists of DEGs using Cytoscape.

**Figure 4:**
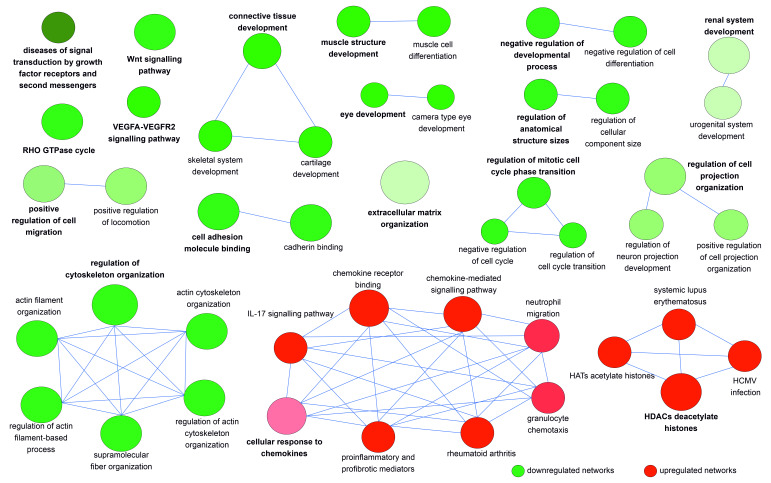
Network representation of enriched GO terms/pathways obtained from the differentially expressed genes in hEDS/HSD. Functionally grouped clusters based on “Biological Process” and “Molecular Function” GO categories and WikiPathways, KEGG and Reactome pathways with terms as nodes linked based on their κ score level = 0.7 through Cytoscape. The GO terms “fusion” and “all experimental evidence” were selected to create the network. Two-sided hypergeometric test and Benjamini–Hochberg statistical correction were applied and only enriched pathways with a *p*-value ≤ 0.01 were selected. Other considered parameters were minimum and maximum tree GO interval values of 3–8, with a minimum number of three genes and 7% of genes selected for GO terms. Different node colors represent different GO categories/enriched pathways: green identify downregulated clusters, red upregulated ones. The increase in green and red color gradient represents higher amounts of the contribution of up- and downregulated genes, respectively. The size of the nodes is indicative of their statistical significance of the terms.

Due to the huge number of nodes and edges deriving from an analysis with 952 DEGs, we applied very stringent statistical criteria that resulted in 18 functionally grouped clusters (Figure 4); the full list of genes involved in the enriched GO terms/pathways is available in Supplementary Table S5.

Among the 16 downregulated clusters, there were several disease-associated signaling pathways including “Wnt signaling”, “VEGFA-VEGFR2 signaling”, and “RHO GTPase cycle” involved in different developmental processes such as “connective tissue development”, “skeletal system development”, “cartilage development”, “muscle structure development”, “eye development”, “renal system development”, and “regulation of anatomical structure size”. The dysregulated expression of several genes encoding growth factors, receptors, second messengers, and transcription factors alongside cell adhesion and cytoskeletal proteins, ECM structural components and ECM-modifying enzymes, also alters a range of cellular functions related to “extracellular matrix organization”, “positive regulation of cell migration”, “negative regulation of cell differentiation”, “cell adhesion molecule binding”, “regulation of mitotic cell cycle phase transition”, “regulation of cell projection organization”, and “regulation of cytoskeleton organization”.

Among upregulated clusters, the most significant group was related to chemokines with enriched GO terms/pathways including “cellular response to chemokines”, “proinflammatory and profibrotic mediators”, “granulocyte chemotaxis”, “neutrophil migration”, “interleukin-17 signaling”, and “rheumatoid arthritis”. The other upregulated network was related to chromatin organization and nucleosome assembly with functional nodes including the pathways “HDACs deacetylate histones”, “HATs acetylate histones”, “systemic lupus erythematosus”, and “HCMV infection”.

### 3.4. qPCR Validation of RNA-Seq Data

Based on these enrichment analyses, we performed qPCR validation of a set of significant DEGs encoding cytokines, related to ECM, cytoskeleton, and chromatin organization, and coding for different growth factors, receptors, and signal transducers by comparing hEDS/HSD vs. control.

**Figure 5:**
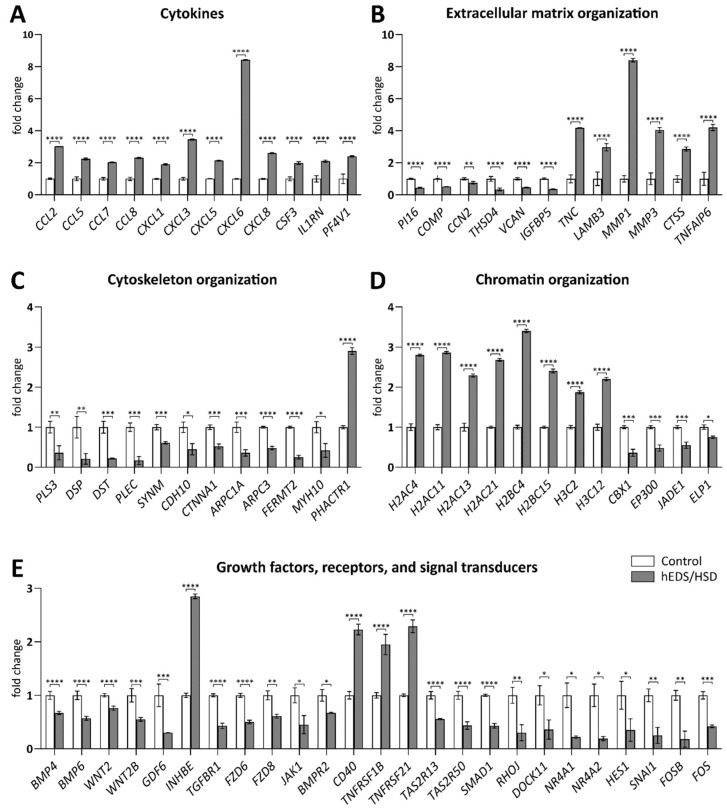
qPCR validation of RNA-seq data. The relative mRNA expression levels of a selection of DEGs encoding cytokines (**A**), involved in ECM organization (**B**), cytoskeleton organization (**C**), chromatin organization (**D**), and coding for different growth factors, receptors, and signal transducers involved in TGFβ, BMP, TNF, Wnt, VEGFA-VEGFR2, and IFN signaling pathways (**E**) were assessed with the 2^−(ΔΔCt)^ method normalized with the geometric mean of different reference genes (*HPRT*, *GAPDH*, *ATP5B*, *RPLP0*, *CYC1*). Bars report average values of triplicates and represent the mean ratio of target gene expression from pooled RNA samples of 20 hEDS/HSD and 20 control cells. Statistical data were obtained with GraphPad Prism 8.0 by applying a two-tailed unpaired *t*-test and expressed as mean ± SEM. * *p* < 0.05, ** *p* < 0.01, *** *p* < 0.001, **** *p* < 0.0001.

Specifically, qPCR confirmed the upregulation of all considered CC and CXC motif chemokine ligands and of the interleukins *CSF3* and *IL1RN* (Figure 5A). In addition, the glycoproteins *TNC*, *LAMB3*, *TNFAIP6*, and the ECM-degrading enzymes *MMP1*, *MMP3*, and *CTSS* were verified to be upregulated and the peptidase inhibitor *PI16*, the glycoproteins *COMP*, *CCN2*, *THSD4*, and *IGFBP5,* and the proteoglycan *VCAN* downregulated (Figure 5B). qPCR also validated the reduced expression of DEGs encoding structural components of the cytoskeleton (*DSP*, *DST*, *MYH10*, *CDH10)* and actin-binding proteins (*PLS3*, *PLEC*, *CTNNA1*, *ARPC1A*, *ARPC3*, *FERMT2)*, as well as the upregulation of the phosphatase and actin regulator *PHACTR1* (Figure 5C). Likewise, all selected genes encoding core histones belonging to the H2A-H2B and H3 families showed increased expression; the nonhistone heterochromatin protein *CBX1* and the histone acetyltransferases *EP300*, *JADE1*, and *ELP1* were downregulated (Figure 5D). Finally, we confirmed the differential expression of several genes involved in TGFβ, BMP, TNF, Wnt, VEGFA-VEGFR2, and IFN signaling pathways and of some members belonging to the bitter taste receptors family. Among secreted growth factors, *BMP4*, *BMP6*, *WNT2*, *WNT2B*, and *GDF6* were downregulated and *INHBE* was upregulated. Similarly, the receptors *TGFBR1*, *FZD6*, *FZD8*, *BMPR2*, *JAK1*, *TASR13*, and *TASR50* were all downregulated, while the TNF receptors *CD40*, *TNFRSF1B*, and *TNFRSF21* showed increased expression. A significant downregulation was also proved for a range of second messengers (*SMAD1*, *RHOJ*, *DOCK11*) and transcription factors (*NR4A1*, *NR4A2*, *HES1*, *SNAI1*, *FOSB*, *FOS*) (Figure 5E).

A subset of these DEGs (10 up- and 10 downregulated) was also verified by qPCR considering hEDS and HSD separately, which confirmed the same trend of differential gene expression of both patients’ groups compared to controls and the substantial lack of differences among hEDS and HSD (Supplementary Figure S5). Overall, the qPCR validation study confirmed the reliability of the RNA-seq data.

## 4. Discussion

Herein, we demonstrated that hEDS and HSD dermal fibroblasts not only share a widespread ECM disarray and a myofibroblast-like phenotype, a combination that is not encountered in other EDS forms and HCTDs in which, however, a disorganization of several ECM structural components might be present [21], but also show a common dysregulated transcriptional signature compared to control fibroblasts. Our molecular data corroborate the current opinion that hEDS and HSD are not distinct entities but clinically overlapping conditions within a single phenotypic continuum, thus reinforcing the awareness of the need for an update of the diagnostic criteria.

Albeit the new criteria were introduced with the expectation to improve phenotyping and clinical diagnoses and to create homogeneous patients’ cohorts for the discovery of the underpinning genetic etiology, several later clinical research emphasized that they are likely inappropriate to clinically differentiate these groups of patients [7,8,9,10,11,12,13,14,15,16,17,18] and, despite multiple attempts, no convincing molecular explanations have been found yet [1,28]. Lack of precision on inclusion criteria for diagnosis, locus heterogeneity, and improper application of the diagnosis in the current clinical scenario, are all contributing to this outward failure.

hEDS and HSD are not limited to JHM and its complaints but are similar complex conditions with wide-ranging systemic (co)morbidities not implemented in the diagnostic criteria, the recognition of which is however crucial for management as they are central determining factors of patients’ reduced quality of life [2,4,6]. The variable and heterogenous phenotypic presentation of either hEDS or HSD, even within single families in which these “labels” often cosegregate, makes a “one-size-fits-all” approach to make a diagnosis very challenging to endorse [7].

In our opinion, the current criteria are limited both by the stringency of the BS and the partly inadequate inclusion of the 12 systemic extra-articular manifestations, several of which also lack real objective definitions (e.g., some cutaneous signs). The criteria also provide equal weight to the comprised multisystemic manifestations despite the common prevalence of some of them in the general population (e.g., piezogenic papules) and the very low frequencies of others (e.g., arachnodactyly, marfanoid habitus, aortic root dilatation) in people with hEDS and HSD [4,7,11,28]. A possible improvement of the diagnostic criteria could be the introduction of a cumulative scoring system based on weighted average of signs/symptoms, as adopted in the revised Ghent criteria for Marfan syndrome [29].

For a forthcoming nosological revision, the multisystemic (co)morbidities to be included and their order of importance should rely on literature data and future clinical research, keeping in mind that they are also frequent in other EDS types and HCTDs [4,6,24]. Future clinical research essential to support clinical diagnosis should also focus on proper practices, methods, and questionnaires for evaluating gJHM including its assessment outside the BS [2,27,30].

Our RNA-seq cohort, selected from a population of about 300 hEDS/HSD patients, is consistent with these views, as it well represents both the characteristic phenotype of hEDS patients and the most typical and frequent phenotypes of individuals with HSD in our clinical experience. Specifically, hEDS patients are most commonly adult females with gJHM, fulfilling criterion 2 principally for the combination feature A plus feature C, and who also show a plethora of comorbidities, some of which could be included in a weighted scoring system. Notably, the most frequent items of features A in hEDS (as well as in HSD) were cutaneous signs, piezogenic papules, and dental crowding/narrow palate; in hEDS, mitral valve prolapse was also common. Concerning HSD, we recruited individuals with a similar assortment of multisystemic signs and symptoms, all showing at least one musculoskeletal manifestation (feature C of criterion 2), without positive family history (feature B of criterion 2), and with the following distinguishing characteristics denoting the main reasons for their HSD exclusion diagnosis in our clinical practice: (i) criterion 1 positive and feature A negative, also known as generalized HSD [3], (ii) criterion 1 negative and feature A positive, and (iii) negative for both criterion 1 and feature A. Patients without any musculoskeletal complaints (feature C negative) fulfilling or not the other items are less frequent in our entire cohort and were thus not recruited for skin biopsy. These latter rare cases are mostly individuals with uncomplicated JHM (i.e., without any pain and/or dislocations), not always fulling criterion 1 (especially males), generally not reaching the five requested items of feature A, and who are almost always family members of symptomatic hEDS or HSD probands. Indeed, in our long-lasting clinical experience as tertiary referral center for EDS, sporadic patients with asymptomatic JHM, representing the mildest end of the phenotypic spectrum of hEDS/HSD, are very unlikely to come to our attention for a clinical evaluation. In a future perspective, we are very interested in enrolling such individuals from the general population to verify the cellular phenotype of their dermal fibroblasts, as well as to investigate the transcriptional profile by RNA-seq. Our findings, emphasizing that most patients with hEDS and HSD should be grouped within the same category, have important consequences from a genetic point of view and in terms of estimated prevalence, since hEDS/HSD is not as rare a condition as expected from hEDS that is still presumed to be inherited in an autosomal dominant pattern [1,2].

However, the spectrum of severity, the heterogeneous presentations of both hEDS and HSD, and the high cumulative incidence of these disorders (1:500) [31], strongly suggest that hEDS/HSD is most likely caused by various genetic changes with an oligogenic if not polygenic inheritance in combination with lifestyle and environmental factors. These opposing possibilities have limited (and still do) the ability of genetic studies to discover causal genes. The ongoing international whole-genome sequencing project HEDGE [32] on about 1000 hEDS patients is expected to identify the underlying genetic etiology, however, if no (or very few) rare pathogenic variants in single genes will be recognized, a genome-wide association study (GWAS) focusing on common variants is the only possible alternative approach. As a drawback, since GWAS may require more than the currently enrolled individuals to obtain significant odds ratios, including the commoner HSD patients in the study population is more than reasonable. In an integrated omics view, the complex dysregulated gene expression pattern and related pathways identified in this work may support the functional interpretation of candidate genomic variants in such large-scale genomic studies.

RNA-seq data revealed the perturbation of many ECM-related processes including cell adhesion, migration, proliferation, and differentiation mainly involving genes encoding growth factors, ECM and cytoskeletal structural components, ECM regulators (especially proteases), and several signal transducers involved in different disease-associated signaling pathways.

**Figure 6:**
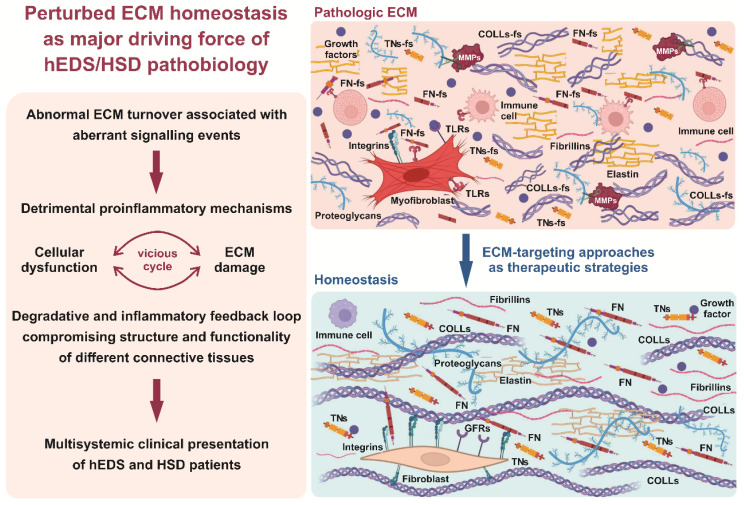
Proposed hEDS/HSD disease model. Schematic representation summarizing the main biological knowledge on molecular mechanisms involved in the hEDS/HSD pathophysiology emerging from the present and our previously published findings [19,22,23]. Abnormal ECM turnover and overproduction of ECM-generated DAMPs act as main triggers in the disease process, sustaining an uncontrolled degradative and proinflammatory vicious cycle affecting homeostasis and integrity of a range of connective tissues, finally leading to the complex clinical manifestation of hEDS/HSD patients. The identification of ECM-related disease drivers may offer ECM-targeting approaches as therapeutic strategies (e.g., doxycycline-mediated MMP inhibition). Abbreviations: COLLs-fs: fragments of collagens; FN-fs: fragments of fibronectin; GFRs: growth factor receptors; MMPs: matrix metalloproteinases; TLRs: Toll-like receptors; TNs-fs: fragments of tenascins. This image was created with BioRender.com.

In a translational view, these data allow to hypothesize that a major disease driver of hEDS/HSD pathogenesis is a detrimental relationship between pathologic ECM, aberrant signaling events, and uncontrolled inflammatory cycle (Figure 6). This pathologic interplay may exacerbate cellular dysfunction and ECM damage, creating a degradative and inflammatory feedback loop that compromises the structure and functionality of different connective tissues, finally leading to the multisystemic clinical presentation of hEDS and HSD patients.

Our assumption agrees with the notion that ECM is not only of physical support for cell and tissue integrity but also dynamically regulates several cellular processes involved in development, homeostasis, and disease progression through its complex of macromolecules and their interaction with cytoskeleton signal transducers [33]. High secretion of a variety of ECM-degrading proteases results in an exaggerated production of different ECM structural constituent fragments that, in turn, disturb the cellular microenvironment by triggering inflammatory reactions [34]. Indeed, these ECM degradation products, also known as danger-associated molecular patterns (DAMPs, or alarmins), are crucial regulators of inflammation pathways since they act as danger sensors by activating cell surface receptors (e.g., Toll-like receptors, TLRs) and downstream proinflammatory signaling cascades. The activation of these pathways leads to the upregulated transcription and secretion of ECM proteases and cytokines, which further increases inflammatory responses also by facilitating recruitment and activation of immune system cells [35]. Consistent with this knowledge and our previous results [19,22], the present gene expression data, showing upregulation of several ECM-degrading enzymes (e.g., *MMP1*, *MMP3*, *CTSS*, *ADAMDEC1*) along with the downregulation of the MMP inhibitor *PI16* [36], further emphasize the pathological contribution of an enhanced proteolytic activity causing a perturbed ECM homeostasis. The upregulation of numerous genes encoding cytokines and the dysregulated expression of several inflammatory-related signal transducers including growth factors, receptors, second messengers, and transcription factors (e.g., *BMPs*, *Wnts*, *TGFβs*, *TNFRs*, *FZDs*, *MAPKs*, *GTPases*, *NR4A1*, *NR4A2*, *HES1*, *SNAI1*, *FOS*, *FOSB*) further supports our proposed disease model. Besides, the involvement of cytoskeleton-dependent mechanotransduction, already described in our previous studies [19,23], is also corroborated by the present work revealing a plethora of downregulated DEGs encoding structural cytoskeleton components (e.g., *DSP*, *DST*, *DMD*, *PLEC*) and actin/cadherin binding proteins (e.g., *ARPC3*, *CDH6*, *CDH10*), some of which (e.g., *AKAP12*, *ARPC1A*, *FERMT2*, *MYH10*, *PLS3*) were also found in our proteome study [23].

Notably, the dysregulated gene expression signature of hEDS/HSD cells is consistent with previous reported gene expression studies in normal meniscus cells, synovial fibroblasts, and articular chondrocytes treated with different proinflammatory factors including cytokines, interleukins, and FN-fs [37,38,39]. These proinflammatory stimuli have been demonstrated to induce transcriptional changes in many genes found differentially expressed also in our patients’ fibroblasts. For instance, the treatment of normal meniscus cells with FN-fs induced the transcriptional modulation of several MMPs, cytokines, TNF family components, and ECM-related genes (*MMP1*, *MMP3*, *CCL2*, *CXCL1*, *CXCL2*, *CXCL8*, *IL1RN*, *CD40*, *TNFRSF1B*, *TNFAIP6*, *CCN2*, *VCAN*, *LAMB3*), all of which were up- or downregulated also in hEDS/HSD cells. Keeping in mind that hEDS/HSD culture medium, capable to induce the pathological phenotype in control cells, contains not only fragments of FN but also of TNs and COLLI (and possibly others) [19,22], the contribution of these DAMPs in the proposed MMPs-mediated degradative and inflammatory feedback loop is more than reasonable. Our proposed disease model, expecting a vicious cycle of DAMPs overproduction and inflammation, also derives from our recently published evidence that doxycycline-mediated MMP inhibition rescues a proper ECM organization in patients’ cells, attenuates their myofibroblast-like features, and prevents the phenotypic switch of control fibroblasts treated with patient cells-derived culture medium [22]. In this view, a pilot clinical trial in hEDS/HSD patients is planned to verify feasibility, safety, and effectiveness of doxycycline treatment.

Transcriptome profiling also revealed upregulated expression of genes encoding different core histones and downregulation of some acetyltransferases involved in chromatin accessibility and transcription regulation. Histones are an example of nuclear DAMPs that may act as proinflammatory mediators contributing to tissue and organ injury in a range of immunological/inflammatory disorders (i.e., rheumatoid arthritis, inflammatory myositides, mixed CTD, and systemic lupus erythematosus) [40]. Further investigations are needed to verify the pathological contribution of histones as DAMPs and/or self-antigens for the formation of autoantibodies [40], as well as a possible epigenetic influence in the hEDS/HSD pathomechanisms.

Based on the present findings, future research may further validate the results of our study. miRNA-seq in patients’ cells is already ongoing to disclose potential mRNA-lncRNA–miRNA interaction networks and attribute a biological significance to the differentially expressed lncRNA identified in the present work.

In addition, translational studies in hEDS/HSD patients’ plasma/serum samples are planned to verify the presence of ECM-derived DAMPs (e.g., FN-, COLLI-, and TNs-fs) and measure proinflammatory/immune mediators and proteins involved in several key processes related to ECM organization, cell differentiation, migration, adhesion, and motility by targeted proteomics through proximity extension assay [41]. These studies are expected to disclose diagnostic biomarkers and related targetable disease pathways that are undeniably needed to revise nosological classification, shorten the hEDS/HSD diagnostic odyssey, and develop therapeutic approaches for patients’ management.

## Data Availability

Most data generated or analyzed during this study are included in this published article and its Supplementary Materials. Additional data and materials are available from the corresponding author on reasonable request, subject to compliance with our obligations under human research ethics. All raw (BAM files) and processed (CHP files) sequencing data were deposited into the Gene Expression Omnibus (GEO) database with the following accession number: GSE218012.

## Supplementary Materials

The following are available online at https://www.mdpi.com/article/10.3390/cells11244040/s1, Figure S1: Detailed representations of the heatmap obtained with the 952 DEGs identified in hEDS/HSD vs. control. Figure S2: Top 10 down-and upregulated GO “Biological Process” categories derived from the 826 downregulated and the 126 upregulated DEGs identified in hEDS/HSD vs. control. Figure S3: Top 10 down-and upregulated GO “Molecular Function” categories derived from the 826 downregulated and the 126 upregulated DEGs identified in hEDS/HSD vs. control. Figure S4: Top 10 down-and upregulated GO “Cellular Component” categories derived from the 826 downregulated and the 126 upregulated DEGs identified in hEDS/HSD vs. control. Figure S5: qPCR validation on a subset of DEGs comparing hEDS vs. control, HSD vs. control, and hEDS vs. HSD. Table S1: Clinical findings of hEDS and HSD patients. Table S2: Differentially expressed genes in hEDS vs. control, HSD vs. control, and in hEDS/HSD vs. control. Table S3: Gene Ontology enrichment analyses of the full list of DEGs identified hEDS/HSD vs. control. Table S4. Gene Ontology enrichment analyses of the 826 downregulated and the 126 upregulated DEGs identified in hEDS/HSD vs. control. Table S5: ClueGO enrichment analysis results.
