# Supplementary material for: RNA-Seq of Dermal Fibroblasts from Patients with Hypermobile Ehlers–Danlos Syndrome and Hypermobility Spectrum Disorders Supports Their Categorization as a Single Entity with Involvement of Extracellular Matrix Degrading and Proinflammatory Pathomechanisms
Source: Cells. 2022 Dec 14;11(24):4040. doi: 10.3390/cells11244040 (PMC9777098; doi:10.3390/cells11244040)
Supplement: Supplementary file 1 [file cells-11-04040-s001.zip › Supplementary materials/Supplementary Figures.pdf]

Supplementary Figures

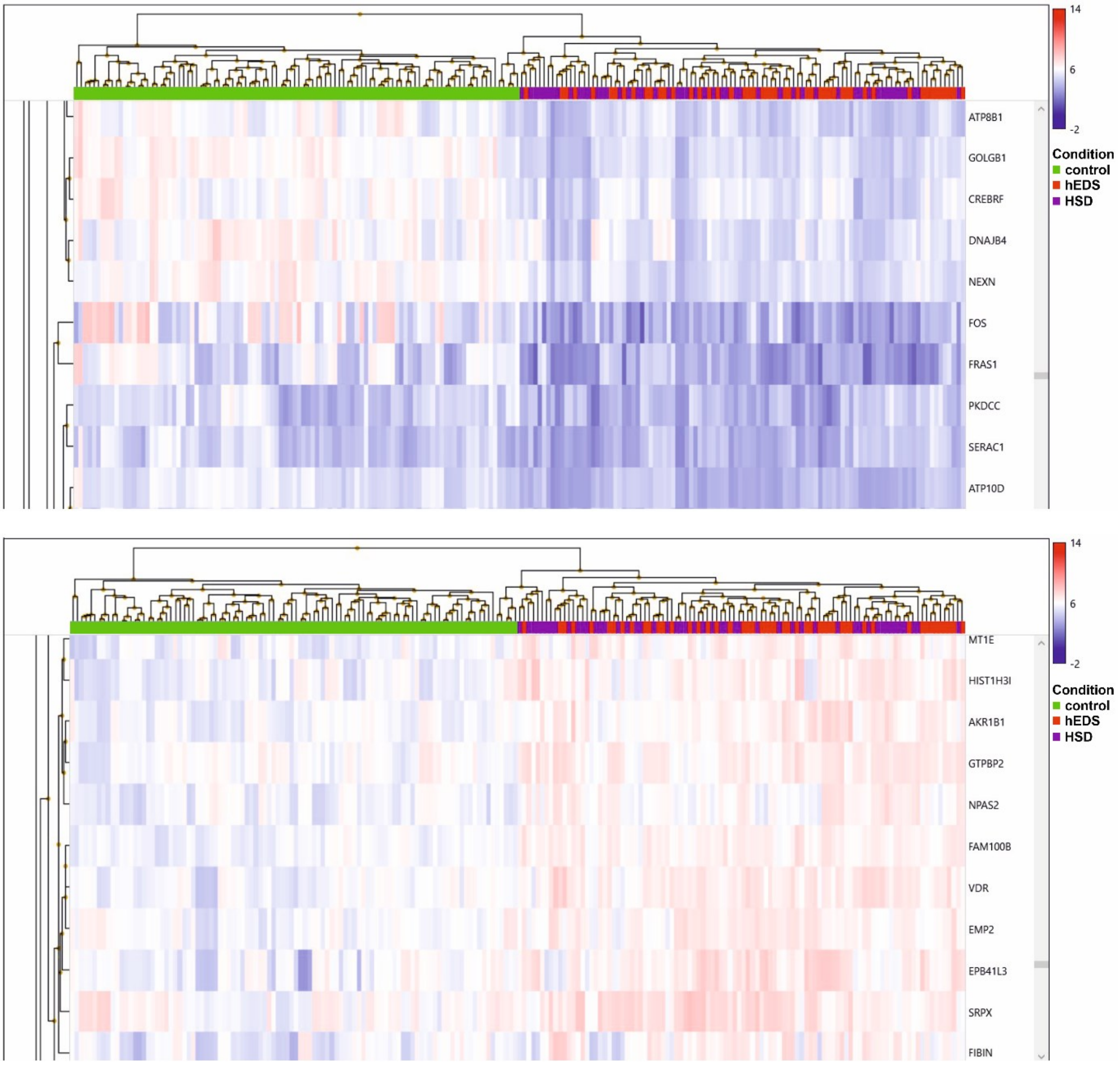

Figure S1: A detail of the gene expression heatmap showing a significant overlap among the expression profiles of hEDS and HSD samples (in red and purple, respectively) compared to control ones (in green). The HCA graph of the 952 DEGs identified in hEDS/HSD vs control (with downregulated genes in blue and upregulated ones in red) was performed by using the Transcriptome Analysis Console 4.0 and applying an ANOVA test with a fold-change threshold of ±1.5 and an FDR-adjusted p-value ≤ 0.01.

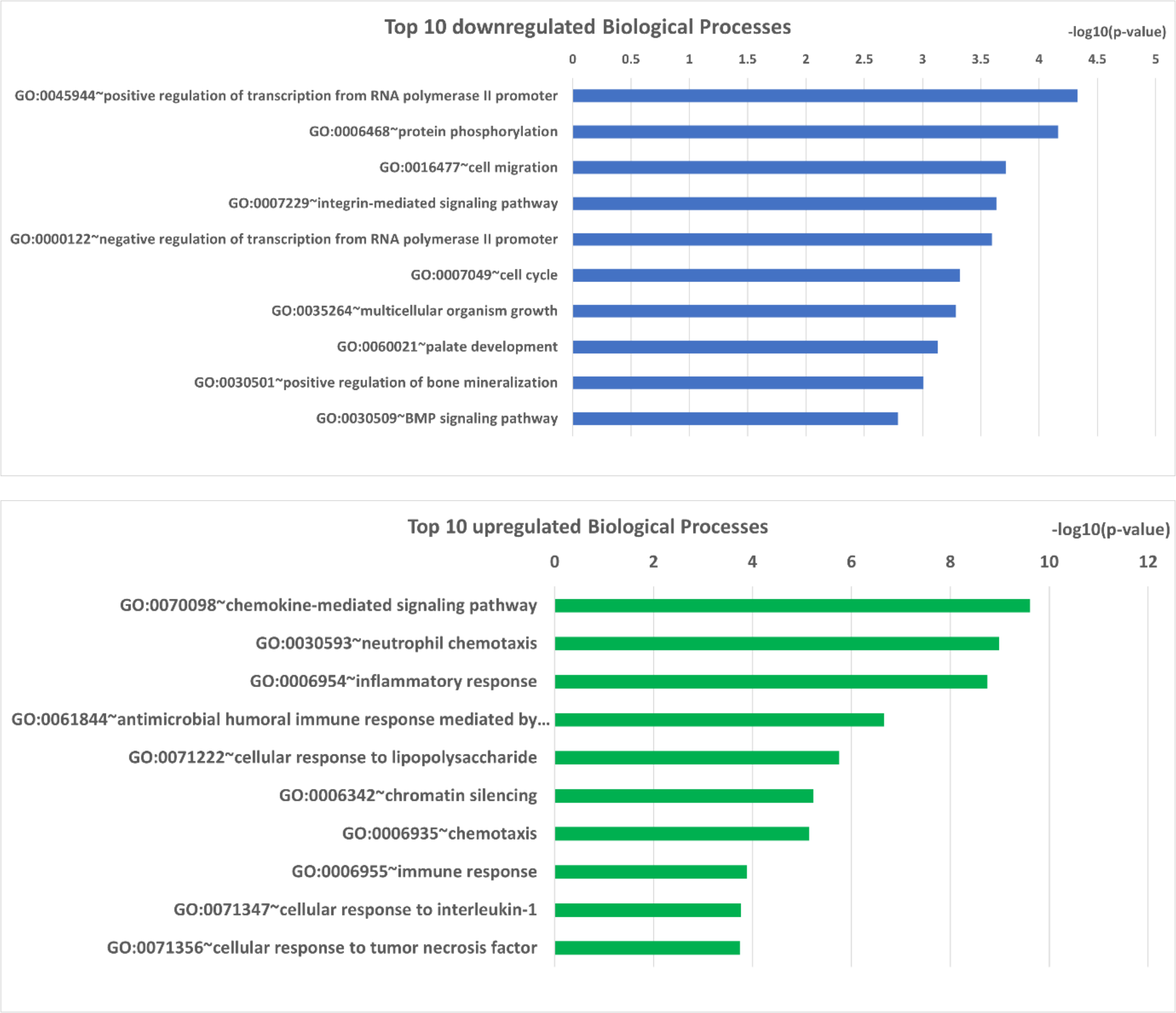

Figure S2: Top 10 down-and upregulated GO "Biological Process" categories derived from the 826 downregulated and the 126 upregulated DEGs identified in hEDS/HSD vs control.

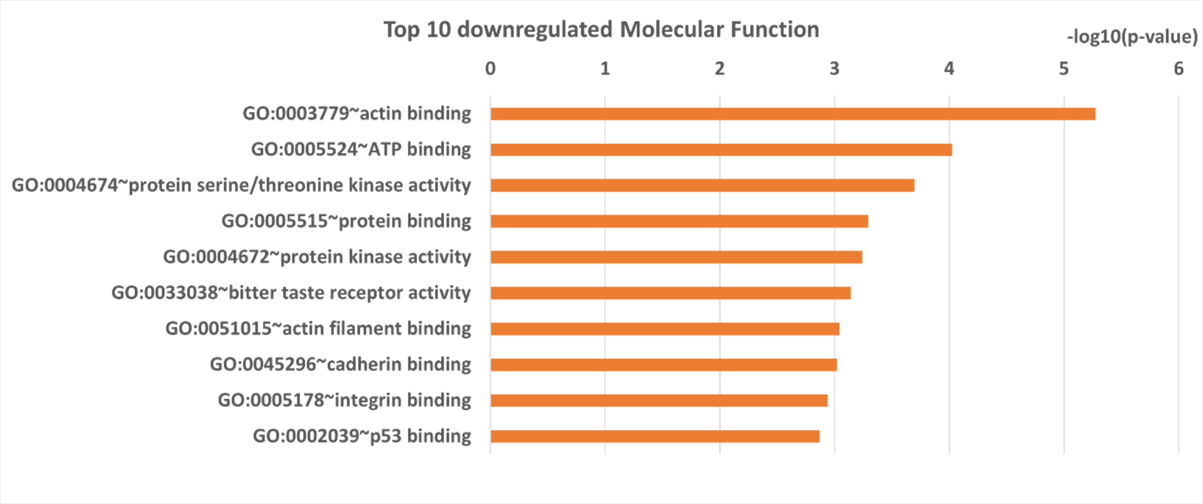

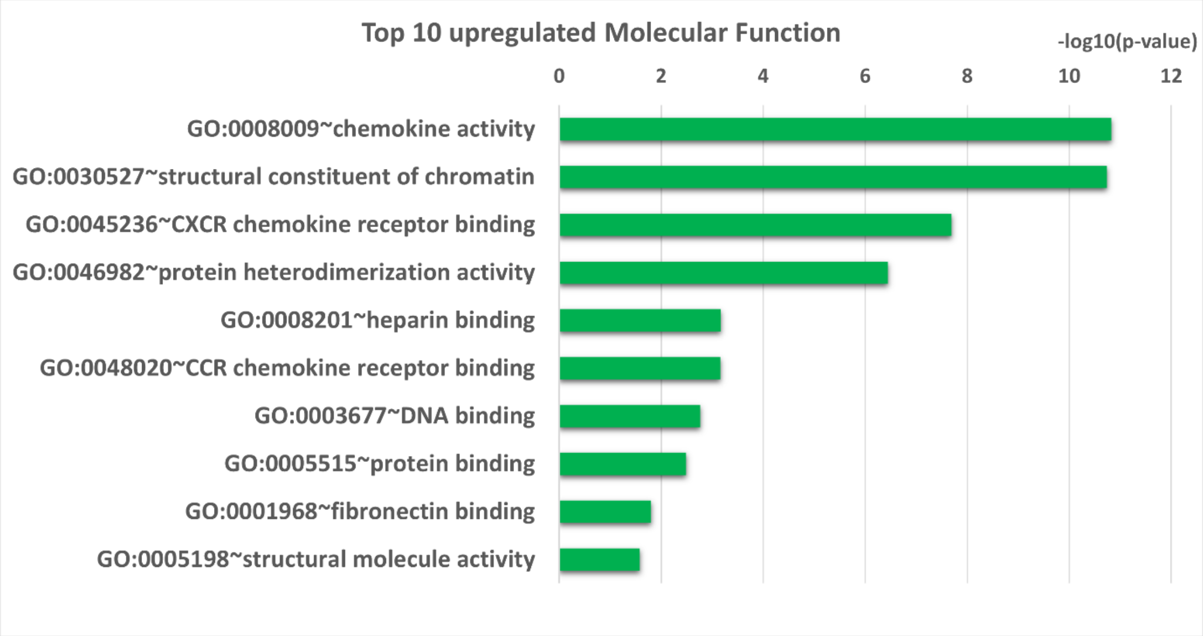

Figure S3: Top 10 down-and upregulated GO "Molecuar Function" categories derived from the 826 downregulated and the 126 upregulated DEGs identified in hEDS/HSD vs control.

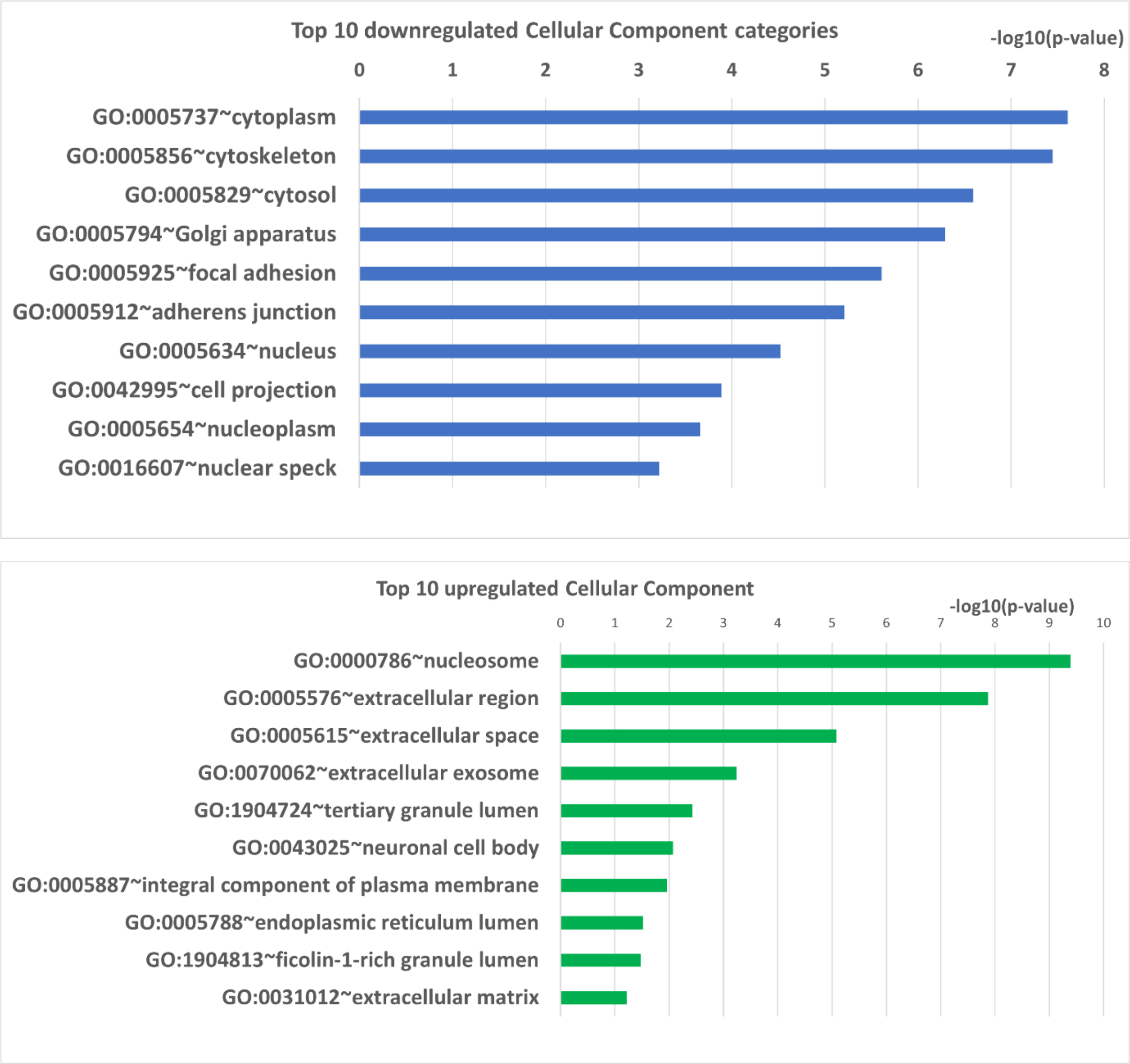

Figure S4: Top 10 down-and upregulated GO "Cellular Component" categories derived from the 826 downregulated and the 126 upregulated DEGs identified in hEDS/HSD vs control.

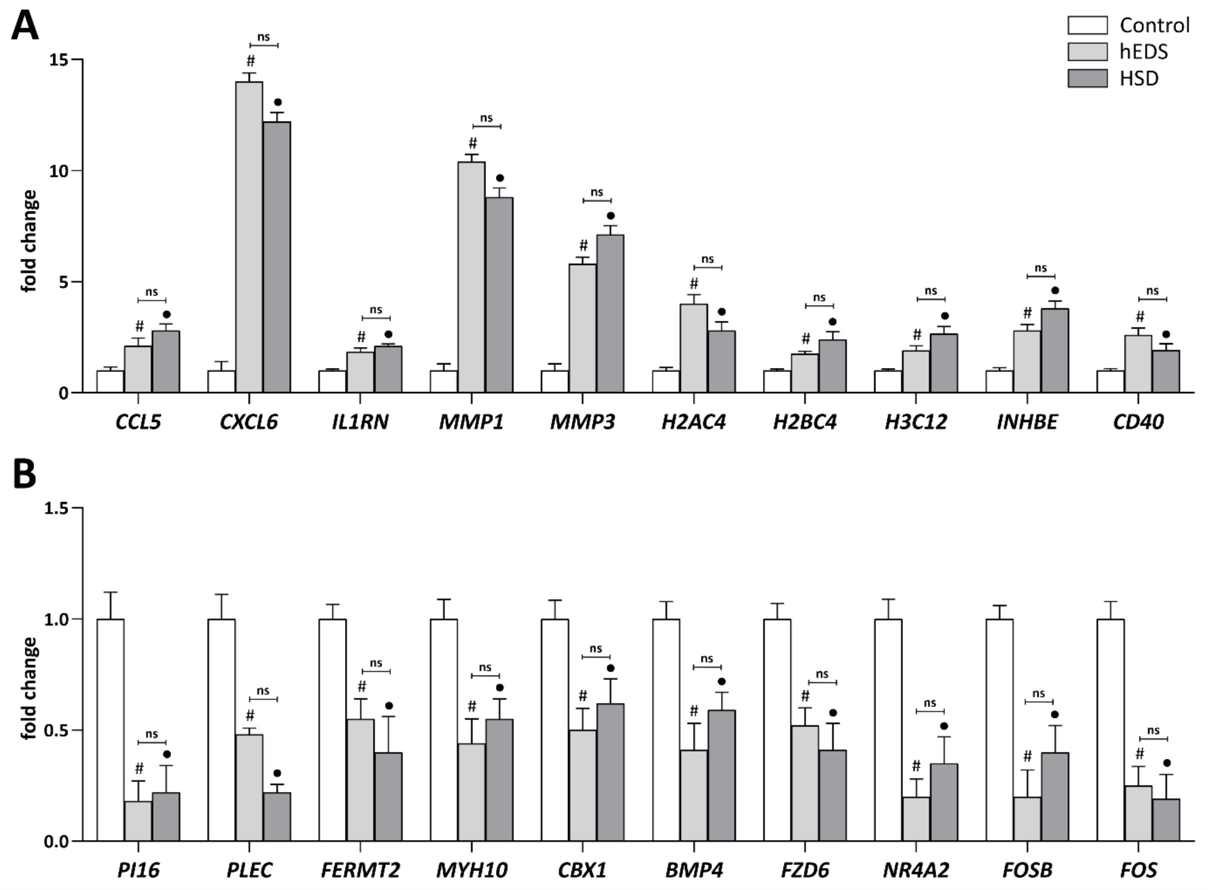

Figure S5. qPCR validation of RNA-seq data. The relative mRNA expression levels of a selection of 10 up- (**A**) and 10 downregulated (**B**) DEGs were assessed with the $2^{-(\Delta\Delta Ct)}$ method normalized with the geometric mean of different reference genes (*HPRT*, *GAPDH*, *ATP5B*, *RPLP0*, *CYC1*). Bars report average values of triplicates and represent the mean ratio of target gene expression from pooled RNA samples of all control, hEDS and HSD cells strains used in the RNA-seq, including the biological replicates (control n=80, hEDS n=40, HSD n=40). Statistical data expressed as mean ± SEM were obtained with GraphPad Prism 8.0 by applying a one-way ANOVA statistical test with multiple comparisons. Only Benjamini-Hochberg FDR-adjusted p-value ≤0.01 were considered. #: significant p-values in control vs hEDS, •: significant p-values control vs HSD, ns: not significant.
